# *Plasmodium* species occurrence, temporal distribution and interaction in a child-aged population in rural Burkina Faso

**DOI:** 10.1186/1475-2875-12-67

**Published:** 2013-02-19

**Authors:** Awa Gnémé, Wamdaogo M Guelbéogo, Michelle M Riehle, Alfred B Tiono, Amidou Diarra, Gustave B Kabré, N’falé Sagnon, Kenneth D Vernick

**Affiliations:** 1 Université de Ouagadougou, Ouagadougou, Burkina Faso; 2 Centre National de recherche et de Formation sur le Paludisme, Ouagadougou, Burkina Faso; 3 Unité de Génétique et Génomique des Insectes Vecteurs, Département de Parasitologie et Mycologie, Institut Pasteur de Paris, Paris, France; 4 Department of Microbiology, University of Minnesota, Minnesota, USA

**Keywords:** Malaria, *Plasmodium*, Species, Infection, Gametocytes, Laye, Burkina faso

## Abstract

**Background:**

Malaria can be caused by five *Plasmodium* species. Due to their higher prevalence, much of the research concentrates on *Plasmodium falciparum* and *Plasmodium vivax*. In Burkina Faso, where *P. falciparum* co-exists with *Plasmodium malariae* and *Plasmodium ovale,* there is not much data about the prevalence of the latter two species across human population. Moreover, interactions between co-infecting *Plasmodium* species are not documented. The aim of the current research is to determine species-specific prevalence and temporal distribution. The potential interactions between co-infecting *Plasmodium* species amongst the child-aged population in Burkina Faso are also discussed.

**Methods:**

The study took place in the Sudanese savannah zone in Burkina Faso in a rural village, Laye. Surveys were conducted during the wet season across four years, 2007 to 2010. Volunteers aged three to 15 years with parental signed consent were enrolled. Ten children per week were screened for any history of pain, fever. Parasitological data were obtained by blood slide processing.

**Results:**

Three sympatric *Plasmodium* species were recorded during this study with an average prevalence of 70.7%. Species temporal distribution showed an increase of *P. malariae* parasite prevalence from 0.9% in 2007 to 13.2% in 2010. Within a season, *P. falciparum* occurred in the overall study period while *P. malariae* and *P. ovale* were highly prevalent after the rainy part of this period. Species-specific infection analysis showed that in a comparison of mono-infections, *P. malariae* gametocyte prevalence and median density were higher than those of *P. falciparum* (88.9% *vs* 34.5% and 124.0 *vs* 40.0 gametocytes/μl, respectively). Likewise, in *P. falciparum* co-infections with *P. malariae* or *P. ovale*, gametocyte prevalence was also higher than in *P. falciparum* mono-infection. However, in *P. falciparum* mixed infection with *P. malariae*, *P. falciparum* gametocyte prevalence and median density as well as asexual form density decreased compared to *P. falciparum* mono-infection while for *P. malariae* mono-infection, only asexual form density significantly vary.

**Conclusion:**

These data revealed high gametocyte prevalence in other *Plasmodium* species than *P. falciparum* with a significant variation of *P. malariae* gametocyte carriers and gametocyte density across years. Molecular tools and entomological studies are needed to highly assess species-specific contribution to malaria transmission.

## Background

Malaria remains a huge burden for human populations living in tropical areas. More than two million malaria cases were recorded in 2010, with the heaviest mortality rates in children living in sub-Saharan Africa [1]. During the past decade, the scale-up of malaria control interventions has resulted in considerable reductions in morbidity and mortality associated with malaria in parts of Africa [2,3]. Despite these efforts, malaria continues to pose a major public health threat in many African countries [1] and recent work suggests that control efforts can be followed by infections rebounding to pre-intervention levels [4]. Successful local strategies for malaria control could be based on malaria surveys, which are an important tool to investigate the impact of the disease on population health status in endemic areas [5]. A primary component for this kind of survey should be the determination of parasite species diversity because optimal control measures are different for the different species of malaria.

Currently, malaria can be caused by five *Plasmodium* species which include *Plasmodium falciparum, Plasmodium malariae, Plasmodium ovale, Plasmodium vivax* and, more recently, *Plasmodium knowlesi*[6]. *Plasmodium falciparum* is the most prevalent in Africa and the most pathogenic of these, but in most malaria endemic regions multiple sympatric species are found and co-infection within individual human hosts or the mosquito vector population is common. In Africa, *P. malariae* is the species most frequently found in sympatry with *P. falciparum*[7]. *Plasmodium* inter-species interactions have been the focus of interest of number of studies [8-11]. The co-infecting species interactions in humans can modify within-host dynamics [12,13] and alter transmission potential [8]. The effect of mixed species infections on clinical outcome has been described as both beneficial [14] and adverse [15]. Indeed, in areas where *P. falciparum* co-occurred with *P. malariae,* field studies reveal that this co-occurrence can reduce disease severity [16,17], give a lower peak of parasitaemia [18] or boost *P. falciparum* gametocyte production [8,9].

In Burkina Faso particularly, where *P. falciparum* co-exists with *P. malariae* and *P. ovale,* there is not much data about the interaction of co-infecting species. In fact, only a few studies even report the prevalence of the last two species across human population [5,19-21]. Most of these studies were done in the past decade before the scale up of interventions such as the availability of artemisinin combination therapy (ACT) or the mass distribution of insecticide-treated bed nets (ITNs). In addition, the country profile reveals an increase in malaria cases from 2008 to 2010 without any information on *P. malariae* and *P. ovale*[1], while some research highlighted a substantial *P. ovale* malaria burden in sub-Saharan Africa [22,23]. Moreover, in Burkina Faso, where the carriage of *P. falciparum* gametocyte is important with a substantial submicroscopic part [24], the role of *P. malariae* or *P. ovale* to this gametocytaemia should be assessed. The aim of the current study was to determine *Plasmodium* species-specific parasite and gametocyte prevalence, their temporal distribution and potential interaction across a child-aged population from a rural village in Burkina Faso.

## Methods

### Study area and period

The study took place in Laye, a small rural village located 30 km north of Ouagadougou, Burkina Faso (12°31’N, 1°46^′^W). This village is situated in a Sudanese savannah zone with one rainy season from June to October with some fluctuation across years. In the village, health care facilities are available and it is also a part of the demographic surveillance system (DSS) used by the malaria research centre of Burkina Faso for epidemiological studies. Residents live by subsistence farming in this Sudanese savannah area with seasonal holo-endemic malaria. Malaria prevalence is highest in the rainy season from June to October and peaks around September. *Plasmodium falciparum* is responsible for the major proportion of malaria infections [20]. Malaria vectors are *Anopheles gambiae* complex members and *Anopheles funestus* groups with other minor vectors [25]. The present survey was conducted in the wet season for four consecutive years from 2007 to 2010. The study was coupled with mosquito larvae sampling with data collection beginning when breeding sites were productive. Thus, the exact time of samples collection was determined by the beginning of the rains and varied across the four sampling years.

### Study population

The study population included children aged three to 15 years. Based on the most recent update of the DSS database, a list of potential children to be enrolled was done each year. Once a week, the households of a computer-generated sample of 10 children were visited by a field worker and a member of the research staff to obtain informed consent for the participation of these children in the study. Only volunteer children with signed consent were brought to the CNRFP (Centre National de Recherche et de Formation sur le Paludisme), the national malaria research centre, and enrolled for parasite detection. In situ, they were asked for any history of pain and examined by a physician. Axillary temperature was taken and a blood sample by finger prick was collected.

### Blood slides collection and parasites counting

The blood slide processing was made from finger-prick blood of each individual. The slides were left to air dry and stained with 5% Giemsa for 35 min. One hundred high-power fields (HPF) were examined, and the number of malaria parasites of each species and stage was recorded. The number of parasites per ml of blood was calculated, assuming 200 white blood cells per high-power field and a fixed white cell count of 8,000/μl. Each blood slide sample was read independently by two microscopists. The mean of the results of both microscopists was taken as the parasite density. In the event of a discrepancy between the two readers, in terms of species, presence or absence of malaria parasites, or if parasite densities differed by more than 30%, a third reader was involved. In this case, the arithmetic mean of the two closest readings was used as the final value for parasite density. A slide was considered negative if no parasite stage were seen after the examination of 100 fields.

### Ethical consideration

The study protocol was reviewed and approved by the ethics committee for biomedical research of the Ministry of Health of Burkina Faso (code N° 2006–032). The study procedures, benefits and risks were explained to the children’s parents or legal guardians and their signed consent was obtained. After each assay, symptomatic subjects were treated with the combination of artemether-lumefantrine (Coartem^®^) according to relevant regulations of Burkina Faso’s Health Ministry. Asymptomatic subjects were followed up by the field worker in the week.

### Meteorological data

The daily rainfall data come from the DGMAC, (Direction Générale de la Météorologie et de l’Aviation Civile), the country meteorological data collection and reporting system. The monthly mean rainfall was use for analysis.

### Data analysis

Comparison of prevalence was done using the Pearson’s Chi square test or Fisher’s exact test with contingency tables. For parasite density, the non-parametric Wilcoxon rank test for two samples or Kruskal-Wallis test for more than two samples was used. Spearman test of correlation was used to assess the link between rainfall and *Plasmodium* density. The potential interaction was assessed by comparing species-specific mono-infection to mixed infection. Statistical analyses were performed in R 2.10.1 using the R Commander Package [26]. *P-value* of 0.05 or lower was considered statistically significant.

## Results

### Study population characterization

Some 830 children (212, 274, 177 and 167 from transmission seasons 2007, 2008, 2009 and 2010, respectively) participated in the study. The mean age in the study group was 8.1 ± 1.7 years. Children of both genders took equal part in this study: 52.9% (males) *vs* 47.1% (females). Across years, gender distribution (Table 1) did not vary significantly (χ^2^ = 0.15, df = 3, p-value = 0.98). On average, across years, 18.8% of the study population presented with fever and 76.3% of these fever cases were coupled with *Plasmodium* infection.

**Table 1:** Study population characteristics

	**Percentage of children per seasons**			
	**2007**	**2008**	**2009**	**2010**
	**(n = 212)**	**(n = 274)**	**(n = 177)**	**(n = 167)**
**Age in years**				
mean (SD*)	9.5 (3.4)	7.6(1.8)	7.7(1.3)	7.6 (1.1)
Median (IQR*)	9.6 (7.1-12.0)	7.4 (6.0-9.3)	7.4 (6.6-8.7)	7.7 (6.7-8.4)
**Genders,%**				
Female	46.7	47.4	48.0	46.1
Male	53.3	52.6	52.0	53.9
**Fever**	21.2	20.1	22.0	10.2

*SD = Standard deviation, IQR = Interquartile Range.

### *Plasmodium* species occurrence and temporal distribution

In the study period, three parasite species: *P. falciparum, P. malariae* and *P. ovale* were identified. Across years, the average *Plasmodium* infection prevalence was 70.7%. *Plasmodium falciparum* was the predominant species with a prevalence of 68.19% followed by *P. malariae* (6.51%) and *P. ovale* (1.08%). Several mixed infections were reported with co-infection of *P. falciparum* and *P. malariae* (4.34%) and co-infection of *P. falciparum* with *P. ovale* (0.72%). No co-infections of *P. malariae* with *P. ovale* were observed, though with their low overall infection prevalence this is not surprising.

In addition, from 2007 to 2010, *Plasmodium* infection prevalence showed slight variation with gametocyte prevalence being significantly different across the four seasons (χ^2^ = 12.8, df = 3, p-value = 0.005). *Plasmodium malariae* infection prevalence was also variable across years (Fisher’s Exact Test, p-value = 3.93e-06) with an increase from 0.9% in 2007 to 13.2% in 2010 (Table 2). As with *P. malariae* overall parasite infection prevalence, gametocyte prevalence also showed a variation across years with higher gametocyte prevalence (14.1%) in 2010. However, *P. falciparum* and *P. ovale* infection prevalence do not significantly vary between the transmission seasons.

**Table 2:** ***Plasmodium *species prevalence across years in the overall population**

		**Seasons**				p-value*
		**2007**	**2008**	**2009**	**2010**	
Parasite Prevalence (%)	Number of children	212	274	177	167	
	All *Plasmodium*	64.2	71.9	71.2	76.6	*0.06*
	*P. falciparum*	63.2	70.4	66.7	72. 5	*0.2*
	*P. malariae*	0.9	5.1	8.5	13.2	*3.93 e-06*
	*P. ovale*	0.5	0.7	1.7	1.8	*0.5*
Gametocyte Prevalence (%)	Number of infected children	136	197	126	128	
	All *Plasmodium*	33.8	36.0	38.1	53.1	*0.005*
	*P. falciparum*	30.9	31.5	26.2	40.6	*0.09*
	*P. malariae*	2.2	3.6	11.1	14.1	*9.5 e-05*
	*P. ovale*	0.7	1.0	1.6	1.6	*0.8*

* p-value tests for variation across years.

**Figure 1:**
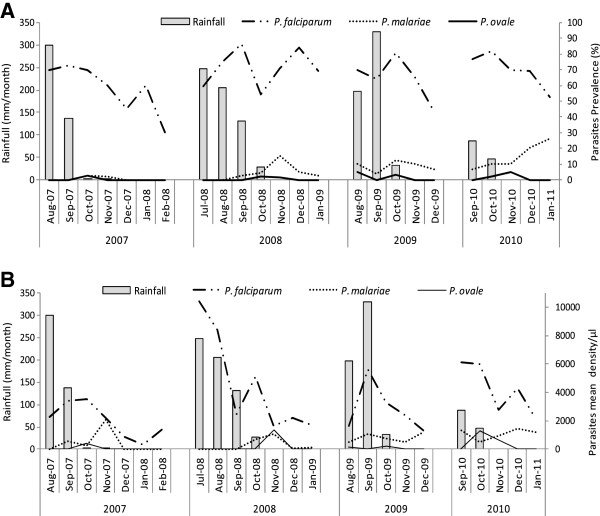
***Plasmodium *species temporal variation across the study period: parasites prevalence (A) and mean density (B).** The rainfall data were obtained from the country’s meteorological data collection and reporting system. *Plasmodium falciparum* reached its maximum prevalence and density in August-October during the rainy season while *Plasmodium malariae* and *Plasmodium ovale* peaked nearer the end of the rainy season.

Within season, species distribution was different with *P. falciparum* being present throughout the study period while *P. malariae* and *P. ovale* often occurred following the rainy part of the study period (Figure 1A). In addition, *P. falciparum* reached its maximum density during the rainy part of the study period, while *P. malariae* and *P. ovale* often peaked nearer the end of this rainy period (Figure 1B). Moreover, Spearman test of correlation, reported a significant positive link between *P. falciparum* density and rainfall (rho = 0.727, p-value = 0.0004). However, *P. malariae* and *P. ovale* density were not significantly correlated with rainfall (respectively rho = −0.153, p-value = 0.53 and rho = 0.013, p-value = 0.96).

### Species characteristics among the infected population

Species-specific mono-infection and mixed infection characteristics are given in Table 3. The median age as well as gametocyte prevalence and median density show significant variation across species infected children (see Table 3). Comparisons of mono-infections characteristics show significant difference for gametocytes prevalence and density (Fisher's Exact Test, p-value = 1.5e-06 and Kruscal-Wallis test, HW = 15.62, df = 2, p-value =0.0004 respectively). Within these mono-infections, the gametocyte prevalence was higher in *P. malariae* infections than in *P. falciparum* infections (88.9% vs. 34.5% respectively, χ^2^ = 22.2, df = 1, p-value = 2.4e-06). The median gametocyte density shows the same trend with higher values being observed in *P. malariae* infections than in *P. falciparum* infections (124.0 vs. 40.0 gametocytes/μl, Wilcoxon rank test, W = 668, p-value = 0.0003). No significant difference was found between *P. ovale* gametocytes carriage or median gametocyte density and either of the other *Plasmodium* species.

**Table 3:** Baseline characteristics of *Plasmodium *species infected children*

	**Age, years, median (IQR)**	**Sex, % male (n/N)**	**Fever, % (n/N)**	**Gametocytes carriers, % (n/N)**	**Gametocytes density, median (IQR)**	**Asexual forms density, median (IQR)**
**PF**	8.0	54.6	21.2	34.5	40.0	1007.5
	(6.5-9.4)	(286/524)	(111/524)	(181/524)	(19–78)	(327–3826)
**PM**	7.6	50.0	5.6	88.9	124.0	874.0
	(7.2-8.2)	(9/18)	(1/18)	(16/18)	(63–390)	(603–1148)
**PO**	10.0	0.0	33.3	66.7	124.5	277.0
	(9.6-12.2)	(0/3)	(1/3)	(2/3)	(122–127)	(219–756)
**PF + PM**	7.2	52.8	13.9	80.6	90.0	1337.5
	(6.3-8.2)	(19/36)	(5/36)	(29/36)	(38–170)	(718–2031)
**PF + PO**	8.0	50.0	16.7	83.3	20.0	6257.5
	(7.4-8.8)	(3/6)	(1/6)	(5/6)	(19–95)	(2692–7476)
***p-value***	*0.04*	*0.5*	*0.3*	*1.7e-12*	*0.0001*	*0.09*

*PF = *P. falciparum* mono-infection; PM = *P. malariae* mono-infection; PF + PM = *P. falciparum* and *P. malariae* co-infection; PO = *P. ovale* mono-infection; PF + PO = *P. falciparum* and *P. ovale* co-infection; IQR = interquartile range.

For age variation, the median age was significantly higher for *P. ovale* mono-infections (10.0 years, IQR, 9.6-12.2) than for *P. malariae* (7.6 years; Wilcoxon rank test, W = 3, p-value = 0.02) or *P. falciparum* (8.0 years; W = 214, p-value = 0.03). Variables such as gender, prevalence of fever, and asexual form median density, did not show significant difference across mono-infections.

Interestingly, significant differences were observed between parasite species mono-infections and their mixed infections for variables such as gametocytes prevalence or density, asexual forms density, and infected children median age. By comparing *P. falciparum* mono-infections to its mixed infections, the study report that gametocyte prevalence and median gametocyte density were lower in mono-infections than co-infections with *P. malariae* (34.5 *vs* 80.6; χ2 = 30.4, p = 3.5e-08 and 40.0 *vs* 90.0; W = 1,690, p-value = 0.002 respectively). However, the median age of infected-children was higher in *P. falciparum* mono-infections than in co-infections with *P. malariae* (7.2 *vs* 8.0; W = 11,430.5, p-value = 0.03).

When looking at *P. falciparum* mixed infection with *P. ovale*, gametocyte prevalence and asexual form density were higher in mixed infections than in *P. falciparum* mono-infections (83.3 *vs* 34.5; Fisher exact test, p-value = 0.02 and 6,257.5 *vs* 1,007.5; W = 691, p-value = 0.02). Other variables did not shown significant variation.

For *P. malariae* and *P. ovale* mono-infections and their mixed infections with *P. falciparum*, no significant difference was found between *P. malariae* mono (n = 18) and mixed infections (n = 36), while *P. ovale* had a higher median density of asexual forms in mixed infections compared to mono-infections (6,257.5 *vs* 277.0; W = 18, p-value = 0.02). Table 4 summarizes the species-specific infection pair comparisons.

**Table 4:** *Plasmodium *species-specific infection comparison significant level *

	**Age, years, Median (IQR)**	**Sex, % male (n/N)**	**Fever, % (n/N)**	**Gametocytes carriers, % (n/N)**	**Gametocytes density, Median (IQR)**	**Asexual forms density, Median (IQR)**
PF vs PM vs PO	0.08	*0.2*	0.2	1.5e-06	0.0004	0.4
PF vs PM	0.8	0.7	0.1	2.436e-06	0.0003	0.5
PF vs PO	0.04	0.09	0.5	0.3	0.07	0.2
PM vs PO	0.02	0.2	0.3	0.4	1.0	0.2
PF vs PF + PM	0.03	0.8	0.3	3.5e-08	0.002	0.5
PF vs PF + PO	0.7	0.8	1.0	0.02	0.7	0.02
PM vs PF + PM	0.1	0.8	0.6	0.7	0.3	0.1
PO vs PF + PO	0.05	0.1	1.0	1.0	0.4	0.002

*PF = *P. falciparum* mono-infection; PM = *P. malariae* mono-infection; PF + PM = *P. falciparum* and *P. malariae* co-infection; PO = *P. ovale* mono-infection; PF + PO = *P. falciparum* and *P. ovale* co-infection; IQR = interquartile range.

### Species characteristic among *Plasmodium* specific-infected children

To assess species interaction, species-specific characteristics in mono- and mixed infections were compared. *Plasmodium falciparum* gametocyte prevalence, gametocyte median density as well as the median asexual form density in mono-infections was higher than those in mixed infections with *P. malariae* (χ^2^ = 4.8, df = 1, p-value = 0.03, W = 879.5, p-value = 0.009 and W = 10,849.5, p-value = 0.03 respectively) (see Table 5)*.* In its co-infection with *P. ovale*, no significant difference was observed for either gametocyte prevalence (33.3% *vs* 34.5%) or gametocyte density (23.5 *vs* 40.0 gametocytes/μl) in comparison with mono-infection (Fisher exact test, p = 1.0 and Wilcoxon rank test, p = 0.2 respectively). However, *P. falciparum* asexual form median density was higher in its co-infection with *P. ovale* (5,563.5 parasites/μl) compared to mono-infection (1,007.5 parasites/μl) (W = 758, p-value = 0.04).

**Table 5:** Species-specific characteristics among mono-infection or mixed infection*

	**Gametocytes carriers**	**Gametocytes density**	**Asexual parasites density**
	**% (n/N)**	**Median (IQR)**	**Median (IQR)**
***Plasmodium falciparum *infection characteristics**			
**PF**	34.5 (181/524)	40.0 (19–78)	1007.5 (327–3826)
**PF + PM**	16.7 (6/36)	16.0 (10–22)	645.0 (200–1277)
**PF + PO**	33.3 (2/6)	23.5 (16–31)	5563.5 (2510–7163)
*PF vs PF + PM, p-value*	*0.03*	*0.01*	*0.03*
*PF vs PF + PO, p-value*	*1.0*	*0.2*	*0.04*
***Plasmodium malariae *infection characteristics**			
**PM**	88.9 (16/18)	124.0 (63–390)	874.0 (603–1148)
**PF + PM**	72.2 (26/36)	99.0 (49–173)	377.5 (132–751)
*PM vs PF + PM, p-value*	*0.3*	*0.5*	*0.003*
***Plasmodium ovale *infection characteristics**			
**PO**	66.7 (2/3)	124.5 (122–127)	277.0 (219–756)
**PF + PO**	83.3 (5/6)	20.0 (19–56)	341.0 (183–733)
*PO vs PF + PO, p-value*	*1.0*	*0.4*	*0.9*

*PF = *P. falciparum* mono-infection; PM = *P. malariae* mono-infection; PF + PM = *P. falciparum* and *P. malariae* co-infection; PO = *P. ovale* mono-infection; PF + PO = *P. falciparum* and *P. ovale* co-infection; IQR = interquartile range.

Among *P. malariae-*infected children, only asexual forms median density shows a significant difference (W = 163, p-value = 0.003) between mono-infections and mixed infections (Table 5). *Plasmodium ovale* does not show any significant variation between mono- and mixed infections.

## Discussion

Three sympatric species, *P. falciparum*, *P. malariae* and *P. ovale* were observed in the study site, with *P. falciparum* being the most prevalent species. *Plasmodium falciparum* infection is widely prevalent across the entire study period with some monthly fluctuation while *P. malariae* and *P. ovale* individually peak at the end of the rainy part of the study period. Similar fluctuation between *P. malariae* and *P. falciparum* densities has been reported by several other studies [19,27]. Offset peaks may be explained by competition between both species and the fact that immune-protection induced by infection with *P. falciparum* does not seem to be effective against *P. malariae*[19]*.* It has also been suggested that *P. malariae* can be found at higher prevalence when transmission and overall infection rates are lower and it is alleviated from the suppressive effect of *P. falciparum*[10,18]. A similar phenomenon may explain *P. ovale* occurrence. This finding revealed a kind of alternation in the relative contribution of each species to the total parasite indexes that occurred in the study area [28].

The significant increase of *P. malariae* prevalence throughout the four-year study period allows speculation on positive selection of this parasite by medication. In fact, in the study site and its surrounding villages many research activities occurred with treatment of symptomatic children [24,29,30] or a trial for intermittent preventive treatment of malaria in children (IPTc) [31,32]. These symptomatic cases are rarely *P. malariae* infected-children because of this species low density. As a result, *P. malariae* remains in circulation despite the treatment and its transmission goes up in the population. More, with the absence of *P. falciparum* due to drug, *P. malariae* prevalence and density goes up because of the suppressive effect of *P. falciparum*[10,18]. In all cases, particular attention should be paid to this secondary parasite. These secondary parasite species should not be ignored particularly as control interventions are planned and evaluated. Entomological studies are also needed to identify anopheline species which breed around the site and assess their vectorial capacity for a better understanding of this increased *P. malariae* prevalence.

In addition, the high proportion of gametocyte carriers and gametocyte density observed among *P. malariae*-infected children during the study period, suggests that specific attention should be paid to this parasite. Indeed, gametocytes are the currency of transmission from human to mosquito for maintaining the malaria transmission cycle. The presence and infectiousness of gametocytes in circulation determines the success of transmission from humans to mosquitoes. For now, *P. malariae* gametocyte density and mosquito infection are not well documented. Nevertheless it is well known that lower *Plasmodium* density as well as submicroscopic density is infectious to mosquitoes [24,33]. Although considered mild, *P. malariae* is known to cause chronic infections which can last for years and might re-occur decades after initial exposure when people have long since left endemic regions [34]. Any level of infection with this species should be taken into account with relevant treatment. Moreover, this study reports a decrease of *P. malariae* asexual forms density and gametocytes prevalence when *P. falciparum* is present. With vaccine leading to the absence of *P. falciparum*, the inhibitory effect on *P. malariae* may disappear. Then, parasitaemia and gametocyte carriage of *P. malariae* could increase and maintain severe malaria infection. Within the context of vaccine being developed against *P. falciparum*[35], this finding presents a great epidemiological issue because the presence of non-falciparum species will still be a serious health concern to the communities at risk of infection.

The current data reveal that *P. falciparum-* and *P. malariae*-specific gametocyte prevalence, gametocyte and asexual form densities were higher in mono-infection as compared to mixed infections. In summary, the implications of these observations may have a profound repercussion in the outcome of disease control. If the simultaneous infection by other species inhibits *P. falciparum*-gametocyte production [36] and if control measures affect and reduce more effectively *Plasmodium* species other than *P. falciparum*, this inhibitory effect may disappear [28]. This finding is in agreement with some previous studies on *P. falciparum*[28,36]. Indeed in Thailand, Price *et al.*[36] reported that mixed infections with *P. falciparum* and *P. vivax* are associated with a reduction in the prevalence of *P. falciparum* gametocytes. In the same way, Marques *et al.*[28] found in Mozambique that *P. falciparum* gametocytes predominated in single infections. In contrast, it has been reported from studies conducted in Columbia and Kenya that *P. malariae* infection enhanced production of *P. falciparum* gametocytes [8,9]. These conflicting results suggest that the relationship between mixed species infections and gametocytaemia may be different under different endemicities [9].

Then, variability in the interactions between species under different transmission intensities, coupled with different sympatric species combinations may contribute to observed differences in the epidemiology and clinical presentations of malaria between endemic regions [10,37].

Finally, prevalences and densities recorded in the current study may underestimate the real part of each *Plasmodium* species. Much study report that parasite prevalence under microscopy is 2.5-fold lower compared to prevalence found with molecular method and this fold difference was consistent across the different transmission levels in individual studies [38-40] or meta-analysis [41]. In light of these results, a longitudinal parasitological survey using both microscopy and molecular tool are necessary to assess species-specific real prevalence for a better understanding of species interaction. An entomological study is also need to evaluate minor species gametocyte infectiousness and their contribution to malaria transmission.

## Conclusion

Three species of malaria parasite are in circulation in the study area, with a fluctuation of *P. falciparum* and other species prevalence. These data revealed high gametocyte prevalence in other *Plasmodium* species than *P. falciparum*. The high proportion of gametocyte carriers and gametocyte density across *P. malariae*-infected children as well as the increase observed across years, presents a great epidemiological issue in the context of a vaccine being developed against *P. falciparum.* The relevance of these data should be taken into account when general control strategies are planned.

## Competing interest

The authors declare that they have no competing interests.

## Authors’ contributions

AG, WMG, ABT, AD participated in data collection and laboratory procedures. AG, WMG, GBK, MMR, N’FS and KDV conceived and designed the experiments. AG, GBK, analysed the data and wrote the manuscript. All authors reviewed and revised the manuscript. N’FS and KDV coordinated and participated to fund acquisition. All authors read and approved the final manuscript.
